# P-58. Evaluation of Treatment Failure in Serratia marcescens, Morganella morganii, and Providencia spp. Bacteremia Treated with AmpC vs. Non-AmpC Stable Antibiotics

**DOI:** 10.1093/ofid/ofaf695.287

**Published:** 2026-01-11

**Authors:** Saipriya Gadiraju, Kristin E Linder, Anastasia Bilinskaya, Lavanya Jitendranath, Tyler Ackley, Rosanna Li

**Affiliations:** Hartford Hospital, Hartford, CT; Hartford HealthCare, Hartford, Connecticut; Hartford Healthcare, Hartford, Connecticut; Hartford Hospital, Hartford, CT; Hartford Hospital, Hartford, CT; Hartford Healthcare, Hartford, Connecticut

## Abstract

**Background:**

The 2024 IDSA guidelines exclude *Serratia marcescens, Morganella morganii*, and *Providencia* species as organisms at moderate-high risk of AmpC production. This study aimed to evaluate clinical outcomes between patients who received non-AmpC stable (N-AMPC) vs. AmpC stable (AMPC) antibiotics in the treatment of bacteremia caused by these organisms.

**Methods:**

This multicenter, retrospective study included adults (≥18 years) admitted between 8/1/2016 and 8/1/2024 with a newly identified monomicrobial bacteremia with either *S. marcescens, M. morganii, or Providencia spp.* which was susceptible to ceftriaxone. The primary outcome, treatment failure, was evaluated as a composite of: escalation of therapy within 30 days, persistent/recurrent infection with the same pathogen within 30 days, and transition to hospice or mortality during admission. Secondary outcomes included hospital length of stay (LOS).

**Results:**

Of the 299 patients included in the primary analysis, 115 and 184 were in the N-AMPC and AMPC group, respectively. *S. marcescens* was the most common organism identified (75/115 (65%) vs 146/184 (78%)) and urinary tract infection was the most common cause of bacteremia (49/115 (43%) vs 43/184 (23%)) in the N-AMPC and AMPC groups, respectively. Baseline characteristics were similar between groups, except for more females and higher median age in the AMPC group. A higher rate of treatment failure was observed in the AMPC group (11/115 (9.6%) vs 36/184 (19.6%), p=0.0208, OR 2.3 (1.13 to 4.78)). 20/115 (23%) and 61/185 (33%) of patients in the N-AMPC and AMPC group required ICU admission, respectively (p=0.0138). Median hospital LOS was 7.50 [1.04-163] days in the N-AMPC and 7.49 [0.17-690.68] days in the AMPC group (p=0.219). Multivariate logistic regression (MLR) analysis showed ICU admission was significantly associated with treatment failure (p=0.0003, OR 0.299 (0.15 to 0.57)).

**Conclusion:**

Treatment failure was significantly higher in the AMPC group. This may be explained by higher acuity, as ICU admission was found to be an independent predictor of treatment failure. This analysis supports the use of N-AMPC stable regimens in low-risk patients with bacteremia caused by *S. marcescens, M. morganii, and Providencia spp.*

**Disclosures:**

All Authors: No reported disclosures

